# Autobiographical Memory and Future Thinking Specificity and Content in Chronic Pain

**DOI:** 10.3389/fpsyg.2020.624187

**Published:** 2021-01-12

**Authors:** Stella R. Quenstedt, Jillian N. Sucher, Kendall A. Pfeffer, Roland Hart, Adam D. Brown

**Affiliations:** ^1^Department of Psychology, The New School for Social Research, New York, NY, United States; ^2^Department of Counseling and Clinical Psychology, Teachers College, Columbia University, New York, NY, United States; ^3^Department of Psychiatry, New York University Grossman School of Medicine, New York, NY, United States

**Keywords:** autobiographical memory, chronic pain, future thinking, mental health, cognition

## Abstract

Chronic pain is associated with high levels of mental health issues and alterations in cognitive processing. Cognitive-behavioral models illustrate the role of memory alterations (e.g., autobiographical memory and future thinking) in the development and maintenance of chronic pain as well as in mental health disorders which frequently co-occur with chronic pain (e.g., anxiety and mood disorders). This study aims to expand our understanding of specific cognitive mechanisms underlying chronic pain which may in turn shed light on cognitive processes underlying pain-related psychological distress. Individuals (*N* = 84) who reported a history of chronic pain and individuals who reported no history of chronic pain (*N* = 102) were recruited from MTurk to complete an online survey including standardized measures of anxiety and depression and two sentence completion tasks that assessed autobiographical memory and future thinking specificity and content. Chi square analyses revealed that participants who endorsed experiencing chronic pain were significantly more likely to recall at least one painful and negative event and to imagine at least one anticipated painful event in their future. Two ANCOVAs were performed to examine the degree to which chronic pain endorsement influenced specificity in memory and future imagining. Individuals with a history of chronic pain and higher levels of depression symptom severity generated autobiographical memories with significantly less specificity; whereas, individuals with a history of chronic pain also generated future autobiographical events with significantly less specificity. In addition, individuals with a history of chronic pain were more likely to generate episodes related to pain when asked to recall the past or imagine the future. Further research is needed to improve our understanding of the etiology of autobiographical memory and future thinking specificity and content in the pathogenesis of mental health conditions in the context of chronic pain.

## Introduction

It is now widely recognized that chronic pain is a global health concern diminishing the wellbeing and functioning of diverse populations. According to the Center for Disease Control’s (CDC’s) analysis of 2016 National Health Interview Survey (NHIS) data, an estimated 20.4% of U.S. adults had chronic pain. Chronic pain places considerable stress on individuals and families and it is a major economic burden to healthcare systems and employers (Dahlhamer et al., 2018). In recent years, a variety of treatments have shown promise in reducing pain severity and subjective distress. In particular, cognitive-behavioral therapy (CBT) has been increasingly employed to help support patients with chronic pain. Clinical trials have indicated that CBT alone or in conjunction with pharmacological interventions have been shown to reduce pain and pain-related distress (Hoffman et al., 2007). CBT appears also has been shown improve quality of life and activities of daily living among diverse forms of pain, including headache, facial pain, and fibromyalgia (Morley et al., 1999; Hoffman et al., 2007; Wetherell et al., 2011). However, many treatment-seeking individuals fail to benefit from available therapeutic interventions (Turk et al., 2011). Untreated or poorly treated chronic pain has been associated with a wide range of negative outcomes, including overall poor health, functional impairment, and lower levels of quality of life (Phillips, 2009; Prefontaine and Rochette, 2013). Research aimed at the characterization and identification of mechanisms associated with chronic pain may help to better elucidate the syndrome and consequently inform effective treatments.

Although chronic pain is a complex syndrome with many potential therapeutic targets, growing evidence suggests that chronic pain is linked with impairments in a number of cognitive domains (McCracken and Iverson, 2001; Moriarty et al., 2011). Strategies designed to help improve cognitive functioning may in turn help to reduce chronic pain symptoms and symptom burden (Hart et al., 2003; Moriarty et al., 2011). Additionally, there is robust evidence indicating high levels of co-occurring mental health disorders among individuals who experience chronic pain. That is, individuals with chronic pain are more likely to experience anxiety, mood, and substance use disorders (Rosenblum et al., 2003; Barry et al., 2009). Pain severity and distress have also been found to exacerbate the severity (Bair et al., 2003) and course of psychiatric symptoms. As such, treatments for chronic pain often incorporate pharmacological and cognitive-behavioral approaches (Turk et al., 2008).

Therefore, individuals with chronic pain may exhibit cognitive alterations similar to those observed across a range of commonly co-occurring mental health disorders. Autobiographical memory is one aspect of cognitive functioning that is frequently altered in individuals with depression (MDD) and in posttraumatic stress disorder (PTSD). Autobiographical memories refer to a class of long-term memories comprised of episodic and semantic content related to one’s personal life story and self-narrative (Rubin, 1998; Conway and Pleydell-Pearce, 2000). Considerable work has found that the ability to recall specific moments from one’s life plays an important role in maintaining a sense of self-continuity and wellbeing (Addis and Tippett, 2008; Bluck and Liao, 2013), facilitates decision making, and fosters social relationships (Bluck et al., 2005). In clinical populations, and in particular in MDD and PTSD, individuals demonstrate difficulty recalling distinct autobiographical memories, a phenomenon referred to as overgeneralized autobiographical memory (OGM; for a review see Moore and Zoellner, 2007). For example, in many studies examining autobiographical memory in people suffering from affective disorders, individuals with MDD or PTSD are more likely to generate memories that take place over several days or that reference repeated events when compared to healthy individuals (Moore and Zoellner, 2007; Williams et al., 2007; Sumner et al., 2010; Lapidow and Brown, 2016). Difficulties in retrieving specific autobiographical memories has also been identified as a risk factor for MDD and PTSD (Moore and Zoellner, 2007; Sumner, 2012). More recently, training programs for memory specificity have emerged confirming that with practice, individuals can increase their autobiographical memory specificity, which in turn, appears to lead to a reduction in psychiatric symptoms (Neshat-Doost et al., 2013).

In line with theoretical models of autobiographical memory which propose that individuals often reconstruct memories to align with current self-views and future goals (Conway and Pleydell-Pearce, 2000), a number of studies have also found that individuals with mental health disorders are more likely to generate autobiographical memory content that reflects the presence of their symptoms. For example, combat veterans with PTSD, compared to those without PTSD, were more likely to recall memories associated with war (Brown et al., 2012), and individuals with prolonged grief were more likely to recall memories of their lost loved one (Maccallum and Bryant, 2008).

In addition to the alterations in specificity and content found in cases of MDD and PTSD, there is now evidence that these same biases are also found when individuals are asked to imagine the future. Converging findings from brain imaging studies, basic behavioral, and clinical research has found that the processes and brain structures that support the retrieval of autobiographical memories overlaps considerably with how one constructs imagined future autobiographical events (Schacter et al., 2007; Botzung et al., 2008; Addis et al., 2009; D’Argembeau, 2012; Benoit and Schacter, 2015). According to the constructive episodic simulation hypothesis, despite human memory being highly error prone, the flexible and malleable system may facilitate the cognitive capacity to recombine elements from the past in order to simulate and pre-experience anticipated future events (Schacter and Addis, 2007). Like autobiographical memory, the ability to imagine future events has been proposed to support a wide range of functions that underlie wellbeing (Williams et al., 2007). Furthermore, studies involving participants with MDD and PTSD found similar autobiographical memory specificity and content alterations in autobiographical memories and imagined future events (Williams et al., 2007).

Less is known about the characteristics and content of autobiographical memory and future imagined events in chronic pain. Early research on the subject found that acute symptoms of pain combined with negative mood led to impaired autobiographical retrieval (Eich et al., 1990; Bryant, 1993). Studies with individuals experiencing chronic pain have also found alterations in autobiographical memory specificity and content; however, these findings are inconsistent. Liu et al. (2014) found that individuals with chronic pain were more likely to recall overgeneralized autobiographical memories and took longer to recall memories than control participants. In contrast, in a sample of individuals with trauma exposure and chronic pain, neither pain severity nor PTSD diagnosis were related to autobiographical memory specificity. Yet, PTSD and pain intensity were related to content, as they were more likely to recall more negatively valenced events (Siqveland et al., 2019). Other studies have demonstrated a link between chronic pain and the recall of more negative memories (Bryant, 1993; Wright and Morley, 1995; Meyer et al., 2015). Unlike, Liu et al. (2014), one study with a small sample of individuals with and without chronic pain found that individuals with chronic pain generated pain memories faster than non-pain memories, suggesting that these memories may be more accessible for those experiencing chronic pain (Wright and Morley, 1995). In addition to the relative dearth of research and inconsistent findings pertaining to the role of autobiographical memory in chronic pain, to our knowledge, no studies have examined how individuals with chronic pain imagine the future.

Despite some of the inconsistences in retrieval latency, overall, these findings indicate that individuals with chronic pain are more likely to recall overgeneralized memories as well as memories comprised of negative or pain-related content. It would be expected, given the common underlying brain processes, that if alterations in specificity, content and valence were present for autobiographical memories, the same patterns should be present for imagined future events. If found that individuals with chronic pain are more likely to imagine less specific and more negatively valenced, pain-related autobiographical future events, future-oriented cognitions may represent a previously undetected mechanism underlying the development and maintenance of the disorder and a promising entry point for intervention.

There were several aims for this study. First, we sought to examine if those with and without self-reported chronic pain differed in autobiographical memory specificity. Second, the content of autobiographical memories was analyzed to identify potential differences in valence and pain-related content. Third, the specificity and content were examined to ascertain whether potential group differences in specificity and content were found in imagined future events.

## Materials and Methods

### Participants

A total of 198 individuals between the ages of 18 to 65 participated in the study. Advertising for the study indicated for participants with and without symptoms of chronic pain. Participants were recruited from Amazon Mechanical Turk (MTurk) and completed the web-based survey on the online platform Qualtrics. This online labor forum is a well-established marketplace for psychological data collection, in which Human Intelligence Tasks (HITs) can be posted by researchers and accessed by individuals who meet specific recruitment parameters (Shapiro et al., 2013). MTurk workers were required to be at a Master’s level (*i.e.*, MTurk workers who demonstrate a high degree of accuracy across a variety of Requesters) and who have also received a 97% approval rating. Only MTurk workers who met these qualifications had access to the survey link. Following protocols employed in similar research (Morasco et al., 2015), participants were excluded from the survey if they self-reported current thoughts of suicidality or other severe mental health disorders such as Bipolar Disorder or Schizophrenia. Individuals were also excluded if they reported a history of head trauma, a history of a neurological disorder, cancer, epilepsy, multiple sclerosis, or Parkinson’s disease. Individuals were required to have English fluency and received $2.50 as compensation after completing the survey. The study was conducted at the New School and was approved by the New School University IRB board.

A total of 12 participants were removed for not completing the study, failing to follow instructions, or for not meeting the aforementioned criteria. A total of 186 participants (57.5% female) completed the study and met all criteria for inclusion. These participants ranged in age from 23 to 65 (*M* = 40.84; *SD* = 10.09). Respondents were allocated into two separate groups by answer the questions, “Are you currently diagnosed with chronic pain?” If they responded “yes” (*n* = 84; 45.2%), they were categorized a chronic pain participant, and if they responded “no” (*n* = 102; 54.8%), they were placed in the non-chronic pain group. Of those who endorsed experiencing chronic pain, 1 (1.2%) participant reported having chronic pain for 1 year or less, 24 (28.6%) 1 to 3 years, 22 (26.2%) 4 to 5 years, 11 (13.1%) 6 to 8 years, 11 (13.1%) 9 to 12 years, and 15 (17.9%) more than 12 years.

### Measures

#### Anxiety and Depression

*Beck Anxiety Inventory* (BAI, Beck and Steer, 1990) and *Beck Depression Inventory-II* (BDI-II, Beck et al., 1996) were used to assess anxiety and depression symptom severity, respectively. The BAI asked participants to endorse how much they have been bothered by symptoms of anxiety, such as an inability to relax or feeling nervous or afraid. The BDI-II asked respondents to endorse statements related to symptoms of depression such as sadness, loss of pleasure, and guilty feelings. Scale scores are a summation of all items with higher scores representing more severe anxiety or depression. Both scales are 21 items in length and displayed excellent reliability (BAI: α = 0.94; BDI-II: α = 0.92).

#### Memory and Future Specificity

Sentence completion tasks were used to assess autobiographical memory and future thinking specificity. The first of which, “Sentence Completion for Events from the Past Test” (SCEPT), was comprised of 11 sentence stems probing for past personal experiences (Raes et al., 2007). The “Sentence Completion for Events in the Future Test” (SCEFT) consists of nine sentence stems probing for possible personal future events (Anderson and Dewhurst, 2009). The prompts required participants to generate a specific event they personally experienced in the past or might experience in the future. In accordance with the suggested guidelines from previous literature, responses were coded for specificity (1 = specific, 0 = categoric, extended, semantic associate, or no response, Chiu et al., 2019). The scoring of the SCEPT and SCEFT was carried out by one of the authors (SQ), blind to condition. Two additional independent raters, blind to condition and the hypotheses of the study, also coded 50% of the data (κ = 0.81). The number of specific responses was totaled to represent memory and future specificity.

#### Pain-Related Content

Each response was evaluated for the presence of negative emotion and pain-related content. If a participant recalled or imagined a negative event in *any* of their memories, they were given an overall score of 1; if none of their responses included a negative event they were given an overall score of 0. Similarly, if the participant recalled or imagined an event related to their pain, they received a score of 1.

## Results

SPSS Version 26 (IBM Corp, 2019) was used for all analyses. Descriptive statistics of measures along with t-tests assessing variables by chronic pain endorsement can be found in Table 1. Bivariate correlations of age, measures of specificity, and clinical measures can be found in Table 2.

**TABLE 1:** Demographic and clinical characteristics of participants.

Variable	Chronic pain (*N* = 84) *M* [%]	No chronic pain (*N* = 102) *M* [%]	*p* [*X*^2^]
Age	43.30	38.79	0.002
% Female	[60.71%]	[54.90%]	[0.637]
BAI	15.18	9.79	0.003
BDI-II	12.96	8.59	0.003
Memory specificity	2.96	4.40	<0.001
Recalled at least 1 painful memory	[27.38%]	[11.76%]	[7.35**]
Recalled at least 1 negative memory	[85.71%]	[73.53%]	[4.13*]
Future specificity	1.49	2.83	<0.001

*p < 0.05; **p < 0.01.

**TABLE 2:** Correlations among age and measures.

Measure	1	2	3	4
1. Age	–			
2. Anxiety	–0.028	–		
3. Depression	–0.011	0.605***	–	
4. Memory specificity	0.048	−0.272***	−0.366***	–
5. Future specificity	–0.030	−0.161*	−0.227**	0.568***

***Correlation is significant at the0.001 level (2-tailed). **Correlation is significant at the0.01 level (2-tailed). *Correlation is significant at the0.05 level (2-tailed). Anxiety was measured by the Beck Anxiety Inventory (BAI). Depression was measured by the Beck Depression Inventory-II (BDI-II). Memory specificity was measured by the number of specific responses on the Sentence Complete for Events from the Past Test. Future specificity was measured by the number of specific responses on the Sentence Complete for Events in the Future Test.

### Demographics

Demographic and clinical measures are displayed in Table 1. The groups did not differ in gender (*p* > 0.05), but the group of individuals with a history of chronic pain were older (*M_*Chronic Pain*_* = 43.30, *M*_*Control*_ = 38.79, *p* < 0.01) and reported higher scores on BDI-II (*p* < 0.01) and BAI (*p* < 0.01) than non-chronic pain group.

### Autobiographical Memory Specificity

Those with chronic pain generated less specific memories and imagined less specific future events than their counterparts without chronic pain (see Table 1). To further assess the degree to which endorsing chronic pain influences memory specificity and memory specificity, two analyses of covariance (ANCOVA) were performed. The first model assessed memory specificity on chronic pain while controlling for depression and anxiety. Group membership (*chronic pain* versus *no chronic pain*) was deviation coded and all prerequisite assumptions for ANCOVA were met; Levene’s test of equality of error variances yielded insignificant results (*F_1_,_184_* = 2.186, *p* = 0.141). The ANCOVA model (*F_3_,_182_* = 15.149, *p* < 0.001; *R*^2^ = 0.200) revealed that group membership (*t* = 3.751, *p* < 0.001; η*_*p*_*^2^ = 0.072) and depression severity (*t* = −3.370, *p* < 0.01; η*_*p*_*^2^ = 0.059) were associated with memory specificity while anxiety severity (*t* = −0.527, *p* = 0.599; η*_*p*_*^2^ = 0.002) was not. These results suggest that those with chronic pain recall less specific memories than those without chronic pain.

### Future Thinking Specificity

The second ANCOVA was identical to the first but instead of memory specificity the dependent variable was future specificity. This model was also significant (*F_3_,_182_* = 13.710, *p* < 0.001; *R*^2^ = 0.184) and found that group membership (*t* = 5.428, *p* < 0.001; η*_*p*_*^2^ = 0.139) was associated with future specificity. Depression (*t* = −1.802, *p* = 0.073; η*_*p*_*^2^ = 0.018) and anxiety (*t* = 0.175, *p* = 0.861; η*_*p*_*^2^ < 0.001), however, were not significant predictors of future specificity. It is important to note that Levene’s test (*F_1_,_184_* = 7.660, *p* < 0.01) was significant meaning error variances were not equal across groups and main effects should be interpreted with caution.

### Autobiographical Memory and Future Thinking Content

The proportion of participants who generated at least one pain-related or negative event can be found in Table 1. A series of chi-squared analyses revealed differences in the generation of content when recalling and imagining personal events between those with and without chronic pain. First, individuals with chronic pain were more likely to recall at least one pain-related (*X*^2^ = 7.35, *p* < 0.01) and negative memory (*X*^2^ = 4.13, *p* < 0.05) in response to the stem-completion task. In fact, 27% of participants in the chronic pain group recalled at least one painful memory and 86% recalled a negative memory; only 12% of those without chronic pain recalled a pain-related memory and 74% recalled a negative memory. Of those who recalled at least one painful memory, chronic pain group members recalled a range of 1–7 painful memories (M = 1.78, SD = 1.38) as compared to a range of 1-4 painful memories (M = 1.42, SD = 0.86) in the control group (*t* = 0.81, *p* > 0.05). In addition, when asked to complete the future-oriented stems, individuals with chronic pain were significantly more likely to imagine scenarios with pain-related content (*X*^2^ = 15.39, *p* < 0.001) but no more likely to imagine negative events (*X*^2^ = 1.06, *p* > 0.05). That is, 19% of participants in the chronic pain group imagined a painful event while only 2% of those without chronic pain imagined a painful event.

## Discussion

As hypothesized, in line with the bulk of previous research, this study found that individuals with chronic pain demonstrated alterations in autobiographical memory recall and future event imagining. In line with previous research, respondents with chronic pain showed a reduction in specificity when remembering past personal memories and imagining anticipated future events. Moreover, respondents with chronic pain also exhibited differences in terms of the content comprising their memories and future imaginings. That is, individuals with chronic pain were more likely to recall pain-related memories and anticipated future events compared to those without a history of chronic pain.

These data converge with and extend previous research on autobiographical cognitive process in relation to chronic pain. First, the findings build on previous studies showing that individuals with chronic pain tend to generate autobiographical memories with less specificity (Liu et al., 2014). The findings provide some support for the possibility that the link between memory specificity and chronic pain may extend beyond the clinical context. Whereas Liu et al. (2014) found an association between chronic pain and less specificity in a sample of individuals enrolled in outpatient care for chronic pain, our participants were self-identified and were not necessarily treatment seeking. Moreover, these results appear to show that differences in specificity by group (chronic pain vs no-chronic pain) may emerge when using neutral stem completions and not just in response to using positive and negative cues as employed in previous research (Liu et al., 2014).

The findings from this study are also consistent with now a very robust body of research showing that autobiographical memory processes, such as episodic specificity, overlap with future thinking in healthy and clinical populations (Gamble et al., 2019; Schacter and Addis, 2019). Future research may benefit from examining whether enhancing specificity in the context of a therapeutic intervention may in turn address some of the pain-related challenges faced by this population. Given the high comorbidity between chronic pain with mental health disorders, and burgeoning research using autobiographical memory and future thinking specificity training to reduce symptoms of depression and improve positive affect (e.g., Neshat-Doost et al., 2013; Hallford et al., 2019), it would be interesting to examine whether increasing specificity can help reduce distress associated with chronic pain.

In addition to specificity, the biases in content aligned with previous research. The greater likelihood of generating a pain-related memory is consistent with other studies showing that people with chronic pain remember more negative content (Bryant, 1993; Wright and Morley, 1995; Meyer et al., 2015; Siqveland et al., 2019). Similar to prior specificity studies, previous studies showing negative biases in memory content have been found primarily in relation to positively and negatively valenced cue-word tasks. This study used neutral stem completions, and so further underscores the negative bias associated with autobiographical memories generated among individuals with chronic pain. Moreover, individuals with chronic pain were more likely to recall and imagine pain-related content. This appears to be line with cognitive models of autobiographical memory which propose that autobiographical memory is motivated to retrieve and reconstruct memories in line with current self-views and future goals (Conway and Pleydell-Pearce, 2000). Other studies with clinical disorders (e.g., complicated grief, PTSD) have shown similar findings, with autobiographical memory and future thinking content reflecting the current concerns of those individuals in the study (e.g., combat veterans with PTSD recalling and imagining events associated with traumatic events, Maccallum and Bryant, 2008; Brown et al., 2013). However, despite these observed biases, it is important to note that the majority of personal memories and future imaginings were not negative or pain-related. That is, 81% of the participants recalled no pain-related memories; only 12 of 186 (6%) participants recalled 2–7 pain-related memories. Further, over 90% of the sample did not imagine a pain-related event in their future. As such, future work with large samples will help to better understand the extent to which individuals with or without chronic pain generate negative or pain-related content when recalling the past or imagining the future.

Also in line with previous research, respondents with chronic pain reported higher levels of depression and anxiety symptoms than healthy controls. Depression and anxiety were both correlated with reduced specificity and appear to be factors inhibiting the generation of specific memories and imagining of specific future events. Individuals with chronic pain responded to sentence prompts with less specificity for both past and imagined future events. Both group membership (chronic pain versus no chronic pain) and depression severity were associated with impaired memory specificity; however, only chronic pain group membership was associated with specificity in future imagining. This hints that comorbid psychopathology is likely not the only mediating factor between chronic pain and OGM. Cognitive impairment (e.g., Moriarty et al., 2011; Liu et al., 2014), affective processes (e.g., Koechlin et al., 2018), and executive functions (Solberg Nes et al., 2009) associated with chronic pain may be directly related to OGM and overgeneralized future imagining. Future research could shed light on these potential mechanisms by examining if cognitive and affective functioning in chronic pain mediates the relationship between chronic pain status and episodic past and future specificity.

Higher somatic burden (i.e., the cumulative experience of and distress related to somatic symptoms such as pain) is significantly associated with depression and anxiety (Gierk et al., 2014). In a recent study, somatic burden was found to partially mediate the relationship between PTSD and perception of cognitive problems; whereas, no direct effect was found between pain severity and perception of cognitive problems (Bartel et al., 2019). It seems plausible that there is a more complexinteraction occurring between pain severity, the somatic burden of pain, accompanying psychological distress (i.e., anxiety and depression), and OGM which is worthy of investigation. Future studies should consider including a measure of somatic burden as well as pain severity.

Several limitations must be noted. The study was conducted online with participants recruited from MTurk. Future work would benefit from in-person experimental designs, such as the inclusion of a pain induction paradigm to test the hypothesis that individuals with chronic pain remember more negative content and may imagine more negative and/or painful future events, when in a painful state. Second, longitudinal studies would also make it possible to examine the extent to which the frequency of alterations in recall and future imagining over time predicts other maladaptive cognitive processes associated with anxiety and depression, e.g., rumination, functional avoidance, and deficits in social problem solving (Bluck et al., 2005; Raes et al., 2005; Williams et al., 2007). Third, the chronic pain group was slightly older than the non-chronic pain group. Given the potential role of age influencing memory specificity, this should be examined in future work. In addition, type of chronic pain was not assessed. Given the heterogeneity of chronic pain conditions (e.g., lower-back, neck, etc.) future work would benefit from exploring whether these biases vary by source of pain. Moreover, the sentence-completion instruments chosen for examining autobiographical memory and future thinking specificity (SCEPT, SCEFT) were selected because of the online format of the study. Yet, the psychometric properties of such measures are less well known than other measures of specificity, such as the Autobiographical Memory Test (AMT; Williams and Broadbent, 1986), in which participants are asked to produce a specific memory in response to positive and negative cue words within a given time limit. Additional research employing other autobiographical memory and future thinking tasks would be important in the validation of these findings.

Importantly, the exact cognitive mechanisms underlying the lack of specificity demonstrated in individuals with chronic pain is yet to be explained. Additionally, future research should examine the potential benefits of addressing future-oriented cognitions in chronic pain as a treatment adjunct for individuals presenting with anxiety or depression comorbid with chronic pain. Furthermore, given the findings that OGM is a risk factor for the onset and course of depression (Williams et al., 2007; Sumner et al., 2010), treatments that target memory specificity and future thinking (Erten and Brown, 2018) should be investigated as an early intervention for those with chronic pain in order to reduce the risk of developing a concurrent mood disorder.

## Data Availability Statement

The raw data supporting the conclusions of this article will be made available by the authors, without undue reservation.

## Ethics Statement

The studies involving human participants were reviewed and approved by New School University IRB. The patients/participants provided their written informed consent to participate in this study.

## Author Contributions

SQ, JS, and AB were involved in the conceptualization and design of the study. SQ and JS collected the data. SQ, JS, AB, KP, and RH were involved in data analysis, interpretation, and manuscript preparation. All authors contributed to the article and approved the submitted version.

## Conflict of Interest

The authors declare that the research was conducted in the absence of any commercial or financial relationships that could be construed as a potential conflict of interest.
